# *Enterococcus faecalis* YM0831 suppresses sucrose-induced hyperglycemia in a silkworm model and in humans

**DOI:** 10.1038/s42003-019-0407-5

**Published:** 2019-05-02

**Authors:** Yasuhiko Matsumoto, Masaki Ishii, Setsuo Hasegawa, Kazuhisa Sekimizu

**Affiliations:** 1 Teikyo University Institute of Medical Mycology, 359 Otsuka, Hachioji, Tokyo, 192-0395 Japan; 2 Department of Microbiology, Meiji Pharmaceutical University, 2-522-1 Noshio, Kiyose, Tokyo, 204-8588 Japan; 3 Molecular Cell Biology Laboratory, Research Institute of Pharmaceutical Sciences, Faculty of Pharmacy, Musashino University, 1-1-20 Shinmachi Nishitokyo-shi, Tokyo, 202-8585 Japan; 4 Genome Pharmaceuticals Institute Co. Ltd., 3-4-5-2D Hongo, Bunkyo-ku, Tokyo, 113-0033 Japan; 5 Pharmaspur Inc., Toyo building, 1-2-10 Nihonbashi, Chuo-ku, Tokyo, 103-0027 Japan

**Keywords:** Metabolic disorders, Animal disease models, Preclinical research, Translational research, Experimental models of disease

## Abstract

Hyperglycemia caused by excessive intake of sucrose leads to lifestyle-related diseases such as diabetes. Administration of a lactic acid bacterial strain to mice suppresses sucrose-induced hyperglycemia, but evidence for a similar effect in humans is lacking. Here we show that *Enterococcus faecalis* YM0831, identified using an in vivo screening system with silkworms, suppressed sucrose-induced hyperglycemia in humans. *E. faecalis* YM0831 also suppressed glucose-induced hyperglycemia in silkworms. *E. faecalis* YM0831 inhibited glucose uptake by the human intestinal epithelial cell line Caco-2. A transposon insertion mutant of *E. faecalis* YM0831, which showed decreased inhibitory activity against glucose uptake by Caco-2 cells, also exhibited decreased inhibitory activity against both sucrose-induced and glucose-induced hyperglycemia in silkworms. In human clinical trials, oral ingestion of *E. faecalis* YM0831 suppressed the increase in blood glucose in a sucrose tolerance test. These findings suggest that *E. faecalis* YM0831 inhibits intestinal glucose transport and suppresses sucrose-induced hyperglycemia in humans.

## Abstract (web summary)

Yasuhiko Matsumoto et al. show that *E. faecalis* YM0831 suppresses sucrose-induced hyperglycemia in silkworms. This bacterial strain also reduces glucose uptake in human Caco-2 cells and suppresses blood glucose levels in humans exposed to a sucrose tolerance test; suggesting a beneficial role for this lactic acid bacteria in sugar metabolism.

## Introduction

The number of patients with type II diabetes continues to increase all over the world^1,2^. A main cause of the onset of type II diabetes is an increase in blood glucose following sugar intake^3^. Therefore, a method to suppress the increase in blood glucose following excess sugar intake may contribute to maintaining a healthy life in humans.

Sucrose is one of the main sweeteners added to various foods and beverages^4,5^. In the intestinal tract, sucrose is degraded to glucose and fructose by α-glycosidase. These sugars are absorbed from the intestinal tract, resulting in increased blood glucose levels^6–8^. The α-glycosidase inhibitors, acarbose and voglibose, inhibit increases in blood glucose after food intake and are used as therapeutic agents for diabetes^9^. Use of these medicines in daily life for healthy people, however, is not recommended due to negative influences, such as abdominal distention and frequent flatulence. Therefore, foods that contain substances that inhibit sucrose absorption in the intestinal tract are desirable^10,11^.

To develop a food that suppresses increases in blood glucose levels after sucrose ingestion, we focused on lactic acid bacteria, which are used to produce fermented foods. Administration of *Lactobacillus rhamnosus* GG strain, a type of lactic acid bacteria, suppresses the increase in blood glucose after sucrose intake in mice^12^. Furthermore, certain lactic acid bacteria strains possess α-glycosidase inhibitory activity^13^.

We previously established diabetes models using silkworms fed a high glucose diet^14–16^ and a method for searching for substances that suppress increases in blood glucose after sucrose ingestion^17^. The increased levels of hemolymph glucose in silkworms caused by sucrose ingestion is suppressed by oral administration of α-glycosidase inhibitors, such as acarbose and voglibose^17^. We also demonstrated that some lactic acid bacteria strains suppress increases in hemolymph glucose in silkworms fed a sucrose-containing diet^17^. Currently, however, there is no evidence that lactic acid bacteria could be used to decrease blood glucose levels in humans after ingestion of a sucrose-containing diet.

In this paper, we describe that the *Enterococcus faecalis* YM0831 obtained by screening using silkworms suppresses increases in blood glucose after sucrose intake in humans. In addition, we show that yogurt produced by the lactic acid bacteria also suppressed an increase in blood glucose after sucrose ingestion.

## Results

### Search for functional lactic acid bacteria using silkworms

**Fig. 1:**
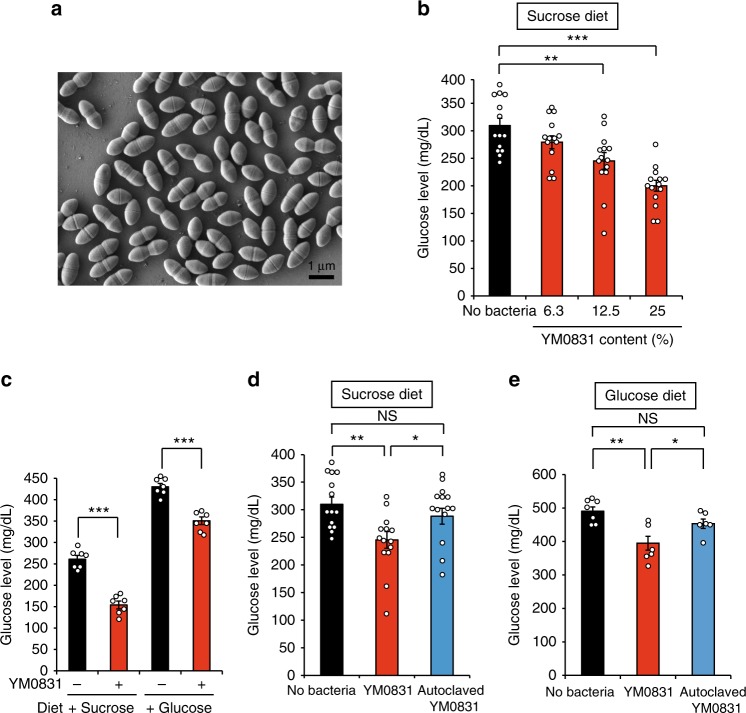
Inhibitory effect of the *E. faecalis* YM0831 against an increase in hemolymph glucose levels in silkworms induced by intake of sucrose or glucose. **a** Electron microscope image of *E. faecalis* YM0831 is shown. Scale bar indicates 1 µm. **b** Silkworms were fed a diet containing 10% (w/w) sucrose with or without *E. faecalis* YM0831 (6.3%, 12.5%, 25% [w/w] in the diet) for 1 h. Glucose levels in the silkworm hemolymph were measured (*n* = 14/group). **c** Silkworms were fed a diet containing 10% (w/w) sucrose or glucose with or without *E. faecalis* YM0831 (25% [w/w] in diet) for 1 h. Glucose levels in the silkworm hemolymph were measured (*n* = 7/group). **d** Silkworms were fed a diet containing 10% (w/w) sucrose with or without *E. faecalis* YM0831 (YM0831, 12.5% [w/w] in diet) or autoclaved *E. faecalis* YM0831 (autoclaved YM0831, 12.5% [w/w] in the diet) for 1 h. Glucose levels in the silkworm hemolymph were measured (*n* = 14/group). **e** Silkworms were fed a diet containing 10% (w/w) glucose with or without *E. faecalis* YM0831 (YM0831, 25% [w/w] in diet) or autoclaved YM0831 (autoclaved YM0831, 25% [w/w] in the diet) for 1 h. Glucose levels in the silkworm hemolymph were measured (*n* = 6–7/group). Data represent mean ± SEM. Statistically significant differences between groups were evaluated using Student’s *t*-test. **P < 0.01. ***P < 0.001. NS: *P* > 0.05

In this study, we first searched for lactic acid bacteria that possess high activity to inhibit the increase in hemolymph glucose in silkworms after sucrose intake. Viable lactic acid bacterial cells were mixed with artificial diet and fed to the silkworms. Out of 50 lactic acid bacteria strains, three strains exhibited suppressive effects on the increase in silkworm hemolymph glucose levels after sucrose intake (Supplementary Table 1). A strain, YM0831, was classified as *E. faecalis* by genetic, morphologic, and biochemical analyses (Fig. 1a, Supplementary Figure 1, Supplementary Tables 2, and 3). The inhibitory effect of *E. faecalis* YM0831 on the increase in hemolymph glucose was dose-dependent (Fig. 1b). We previously reported the inhibitory effects of the α-glycosidase inhibitors acarbose and voglibose against sucrose-induced hyperglycemia in silkworms^17^. We performed an experiment to simultaneously compare the effects of *E. faecalis* YM0831 with those of the α-glycosidase inhibitors acarbose and voglibose (Supplementary Figure 2). Our results demonstrated that sucrose-induced hyperglycemia in silkworms was inhibited by the addition of *E. faecalis* YM0831 at 16% of the dietary weight, but not at 4% of the dietary weight (Supplementary Figure 2). On the other hand, sucrose-induced hyperglycemia in silkworms was inhibited by the addition of acarbose and voglibose at only 1% and 4% of the dietary weight, respectively, but not at 0.25% dietary weight (Supplementary Figure 2). *E. faecalis* YM0831 exhibited an inhibitory effect after intake of both sucrose and glucose against the increase in the hemolymph glucose levels in silkworms (Fig. 1c). When the lactic acid bacteria were autoclaved, no activity to suppress the increase in hemolymph glucose after sucrose intake was observed (Fig. 1d). The activity of *E. faecalis* YM0831 to suppress the increase in the silkworm hemolymph glucose levels after glucose intake was also reduced by autoclaving the lactic acid bacteria (Fig. 1e).

### Inhibitory effect of *E. faecalis* YM0831 on glucose transport

We established an experimental system to examine the inhibitory mechanism of *E. faecalis* YM0831 on sugar absorption in the silkworm intestinal tract. Sucrose solution was added into the lumen of the isolated silkworm intestinal tract and the glucose concentration outside the intestinal tract was measured (Supplementary Figure 3a). The glucose concentration outside of the intestinal tract increased in a time-dependent manner (Supplementary Figure 3b). This increase was inhibited by adding acarbose, an α-glycosidase inhibitor, inside the intestinal tract (Supplementary Figure 3b). The results suggest that sucrose was degraded into glucose and fructose by α-glycosidase, which was present in the silkworm intestinal tract, resulting in translocation of glucose to the outside of the intestinal tract. In the sugar transport system using isolated silkworm intestine, the addition of *E. faecalis* YM0831 cells to the sucrose solution inhibited the increase in the glucose concentration outside of the intestine (Supplementary Figure 3c). We demonstrated that the *E. faecalis* YM0831 did not injure the cells of the isolated intestinal tract of silkworm by measuring the activity of mitochondrial dehydrogenase in the intestinal cells (Supplementary Figure 3d). The results suggest that *E. faecalis* YM0831 inhibits either or both the degradation of sucrose by α-glycosidase and/or the transport of glucose through the intestinal membrane.

**Fig. 2:**
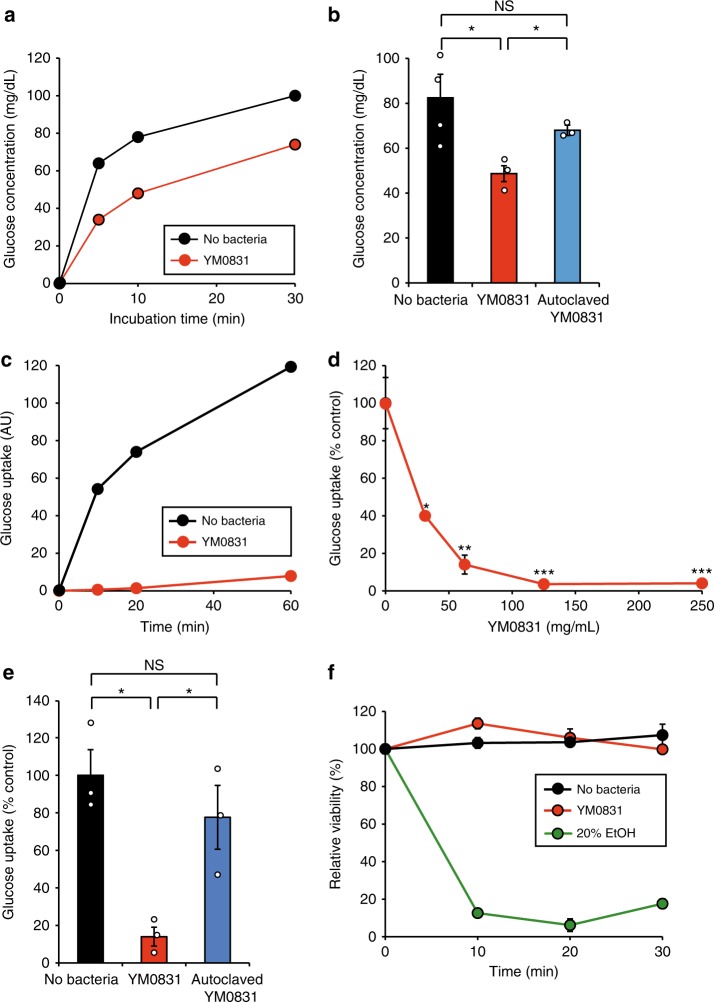
Inhibitory effect of *E. faecalis* YM0831 on glucose transport in the silkworm intestinal tract using isolated intestine and glucose uptake by Caco-2 cells. **a** Glucose solution with or without added *E. faecalis* YM0831 (250 mg wet weight/ml) were enclosed in isolated silkworm intestinal tract. The intestinal samples were incubated in PBS at 27 °C. Glucose levels outside of the intestine were determined. **b** Glucose solution with or without added *E. faecalis* YM0831 (YM0831, 250 mg wet weight/ml), or glucose solution with added autoclaved *E. faecalis* YM0831 (autoclaved YM0831, 250 mg wet weight/ml) were enclosed in isolated silkworm intestinal tract. Intestinal samples were incubated in PBS at 27 °C for 10 min. Glucose levels outside of the intestine were determined. *n* = 3–4/group. **c** *E. faecalis* YM0831 (62.5 mg wet weight cells/ml) was added in the uptake system of 2-NBDG in Caco-2 cells and fluorescence uptake by the Caco-2 cells was measured over time. **d** Various numbers of *E. faecalis* YM0831 were added in the uptake system of 2-NBDG in Caco-2 cells and fluorescence uptake by the Caco-2 cells was measured. *n* = 3/group. **e** *E. faecalis* YM0831 (62.5 mg wet weight cells/ml) or autoclaved *E. faecalis* YM0831 (autoclaved YM0831, 62.5 mg wet weight cells/ml) was added in the uptake system of 2-NBDG in Caco-2 cells and fluorescence uptake by the Caco-2 cells was measured. *n* = 3/group. **f** Viability of Caco-2 cells following the addition or absence of *E. faecalis* YM0831 (62.5 mg wet weight cells/ml) or addition of 20% ethanol solution was measured. *n* = 3/group. Data represent mean ± SEM. Statistically significant differences between control and groups in the presence of samples were evaluated using Student’s *t*-test. **P < 0.01, ***P < 0.001, NS: *P* > 0.05

Next, we investigated the effect of *E. faecalis* YM0831 on glucose transport by adding glucose solution into the lumen of the isolated silkworm intestinal tract. In the experimental system using glucose, the effect of degradation by α-glycosidase can be excluded. When glucose solution was placed inside sections of isolated silkworm intestinal tract, the glucose was transported out of the intestinal tract in a time-dependent manner (Fig. 2a). Adding *E. faecalis* YM0831 cells to the intestinal tract decreased the amount of glucose transported outside the intestinal tract (Fig. 2a). Replacement of the *E. faecalis* YM0831 cells with an autoclaved cell fraction reduced the glucose transport inhibitory effect (Fig. 2b). These results suggest that *E. faecalis* YM0831 inhibits glucose transport in the silkworm intestine.

Caco-2 cells are derived from human intestinal tract, and a method for quantifying glucose uptake into the cells has been established^18^. *E. faecalis* YM0831 cells inhibited glucose uptake by Caco-2 cells (Fig. 2c, d). The inhibitory effect of *E. faecalis* YM0831 cells on glucose uptake was decreased by autoclaving the cells (Fig. 2e). A cell viability assay using WST-1 demonstrated that the *E. faecalis* YM0831 cells were not cytotoxic against Caco-2 cells (Fig. 2f), indicating that the inhibitory effect of the *E. faecalis* YM0831 on glucose uptake was not due to cytotoxic effects of the lactic acid bacteria. These findings suggest that *E. faecalis* YM0831 inhibits glucose uptake into human intestinal epithelial cells.

### Mechanism of suppressive effect of *E. faecalis* YM0831

As the mechanism of action of *E. faecalis* YM0831 to suppress the blood glucose increase following ingestion of sucrose, we considered two possibilities: (i) consumption of sugars by *E. faecalis* YM0831 and (ii) inhibition of glucose uptake by intestinal cells by a substance secreted from *E. faecalis* YM0831. We tested whether *E. faecalis* YM0831 survival is necessary for its inhibitory effect on the blood glucose increase after sucrose intake in silkworms. The viable cell number of *E. faecalis* YM0831 was decreased to <10^−7^ fold by heat-treatment at 80 °C for 15 min (Supplementary Figure 4a). The heat-killed *E. faecalis* YM0831 inhibited sucrose-induced hyperglycemia in silkworms (Supplementary Figure 4b). The heat-killed *E. faecalis* YM0831 also inhibited glucose transport in silkworm intestine and glucose uptake by Caco-2 cells (Supplementary Figure 4c, d). Substances inactivated at 121 °C, but not at 80 °C, seem to be responsible for these effects.

Furthermore, we found that a soluble fraction obtained by sonication of *E. faecalis* YM0831 cells has an inhibitory effect against glucose uptake by Caco-2 cells (Supplementary Figure 5). The result suggests that a soluble substance produced by *E. faecalis* YM0831 inhibits glucose uptake in Caco-2 cells.

**Fig. 3:**
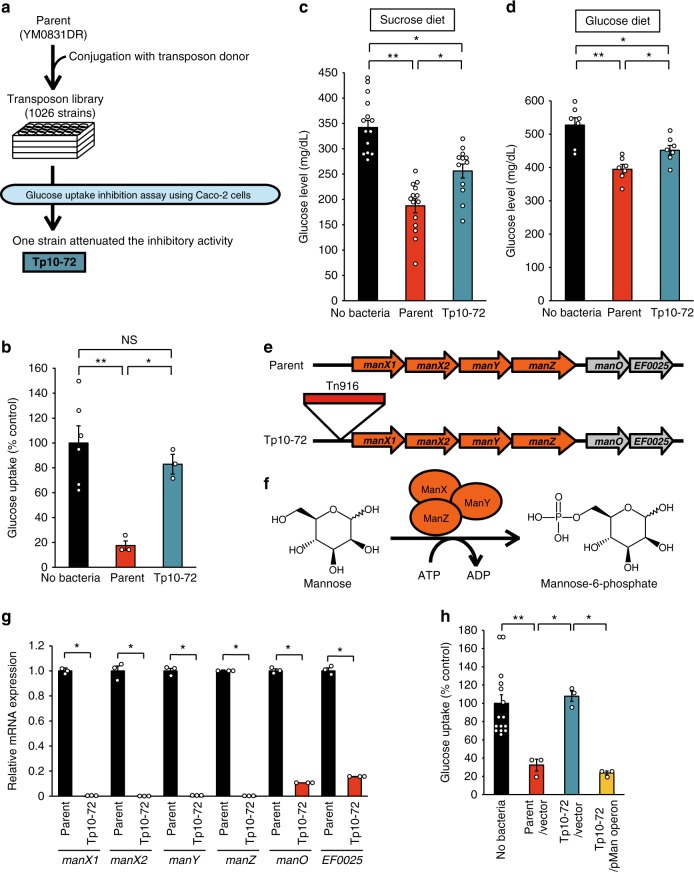
Characterization of a transposon mutant with attenuated inhibitory activity of *E. faecalis* YM0831 against glucose uptake by Caco-2 cells. **a** Experimental scheme of the screening to obtain a transposon mutant with attenuated inhibitory activity of *E. faecalis* YM0831 against glucose uptake by Caco-2 cells. **b** Decrease in inhibitory activity of *E. faecalis* YM0831 transposon mutant (Tp10-72) against glucose uptake by Caco-2 cells. *E. faecalis* YM0831DR (parent, 62.5 mg wet weight cells/ml) or Tp10-72 (Tp10-72, 62.5 mg wet weight cells/ml) was added in the uptake system of 2-NBDG in Caco-2 cells and fluorescence uptake by the Caco-2 cells was measured. *n* = 3–6/group. **c** Decrease in inhibitory activity of Tp10-72 against sucrose-induced hyperglycemia in silkworms. Silkworms were fed a diet containing 10% (w/w) sucrose with or without *E. faecalis* parent (YM0831DR, 12.5% [w/w] in diet) or Tp10-72 (Tp10-72, 12.5% [w/w] in the diet) for 1 h. Glucose levels in the silkworm hemolymph were measured (*n* = 12–14/group). **d** Decrease in inhibitory activity of Tp10-72 against glucose-induced hyperglycemia in silkworms. Silkworms were fed a diet containing 10% (w/w) glucose with or without *E. faecalis* YM0831DR (parent, 12.5% [w/w] in diet) or Tp10-72 (Tp10-72, 12.5% [w/w] in the diet) for 1 h. Glucose levels in the silkworm hemolymph were measured (*n* = 7/group). **e** Inserted region of transposon Tn916 in Tp10-72 genome determined by whole genome sequencing analysis. **f** Functions of ManX, ManY, and ManZ coded by the *man* operon. **g** Decreases in mRNA amounts of genes in the *man* operon in TP10-72 revealed by RT-PCR analysis. **h** Complementation of decreased inhibitory activity of TP10-72 on glucose uptake by Caco-2 cells. *E. faecalis* YM0831DR/pND50 (parent/vector, 70 mg wet weight cells/ml), Tp10-72/pND50 (Tp10-72/vector, 70 mg wet weight cells/ml), or Tp10-72/pMan operon (Tp10-72/pMan operon, 70 mg wet weight cells/ml) were added in the uptake system of 2-NBDG in Caco-2 cells and fluorescence uptake by the Caco-2 cells was measured. *n* = 3–15/group. Data represent mean ± SEM. Statistically significant differences between groups were evaluated using Student’s *t*-test. *P < 0.05; **P < 0.01; ***P < 0.001

Next, to determine if the inhibitory activity of *E. faecalis* YM0831 against glucose uptake by Caco-2 cells is necessary to suppress sucrose-induced hyperglycemia, we isolated an *E. faecalis* YM0831 mutant with decreased inhibitory activity against glucose uptake by Caco-2 cells (Fig. 3a). In this study, we isolated a YM0831DR strain resistant to both rifampicin and kanamycin from *E. faecalis* YM0831 as a parent strain and constructed a library of transposon insertion mutants (Fig. 3a). Among 1026 transposon mutants, Tp10-72 was identified as having decreased inhibitory activity against glucose uptake by Caco-2 cells (Fig. 3a, b). The inhibitory activity of Tp10-72 against sucrose-induced and glucose-induced hyperglycemia in silkworms was also lower than that of the parent strain YM0831DR (Fig. 3c, d). These results demonstrate that the function of the transposon-inserted region-related genes is required for both the inhibitory effect against glucose uptake by Caco-2 cells and the inhibitory effect against sucrose-induced hyperglycemia in silkworms. Our findings indicate that the inhibitory activity of *E. faecalis* YM0831 against glucose uptake by Caco-2 cells underlies its activity to suppress sucrose-induced hyperglycemia.

To identify the transposon insertion site of Tp10-72, we performed genome sequencing analysis. A transposon was inserted into the promoter region of the genes encoding *man* operon in the genome (Fig. 3e). The *man* operon involves genes encoding components of an enzyme synthesizing mannose-6-phosphate from mannose (Fig. 3f). The amount of mRNA of the genes contained in the *man* operon in Tp10-72 were markedly lower than that in the parent strain (Fig. 3g). Furthermore, the decrease in the inhibitory activity against glucose uptake by Caco-2 cells in Tp10-72 was complemented by introducing a plasmid containing the *man* operon region into Tp10-72 (Fig. 3h). The results demonstrated that *E. faecalis* YM0831 inhibited glucose uptake by Caco-2 cells through the expression of genes contained in the *man* operon.

### Comprehensive genomic analysis of the *E. faecalis* YM0831

**Fig. 4:**
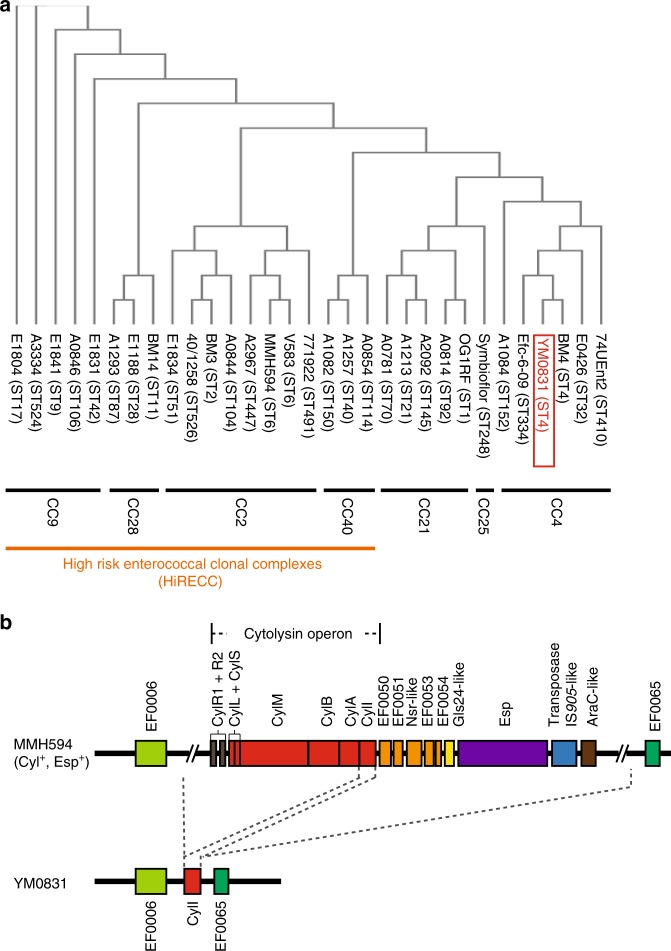
Comparative genome analysis between the *E. faecalis* YM0831 and high-risk strains. **a** The phylogenic tree of the *E. faecalis* YM0831 and strains analyzed for sequencing (ST) and clonal complex (CC) are shown. HiRECC high-risk enterococcal clonal complexes. **b** Comparative gene analysis regarding the presence of cytolysin and Esp contained in the *Enterococcus faecalis* pathogenic island (*37*) in the YM0831 with the high-risk strain, MMH 594

*E. faecalis* is widely used to produce fermented foods such as yogurt. On the other hand, the bacteria cause endocarditis and urinary tract infection in patients with reduced immunity^19,20^. Pathogenic strains can be distinguished from non-pathogenic strains by sequence analysis of chromosomal DNA^21,22^. *E. faecalis* strains are classified based on the sequences of seven genes: *zwf*, *gap-2*, *pstS*, *glcK*, *aroE*, *xpt*, and *ygiL*^21,22^. Based on the sequence obtained from whole genome analysis of the *E. faecalis* YM0831, we classified this strain as ST4 (Fig. 4a). Phylogenetic tree analysis revealed that the *E. faecalis* YM0831 did not belong to CC2, CC9, CC28, or CC40, which are classified as high-risk enterococci groups. The Symbioflor strain is used in yogurt manufacturing and medicine^23,24^. The phylogenic tree analysis indicated that *E. faecalis* YM0831 was close to Symbioflor (Fig. 4a).

*E. faecalis* strains clinically isolated as causative bacteria of infections have a pathogenic island containing genes encoding pathogenic factors, such as cytolysin and Esp^25,26^. CylL and CylS are active effecter proteins of cytolysin. CylA and CylM are necessary for activation of cytolysin. CylB is a transporter of cytolysin. These proteins contribute to the cytotoxicity of *E. faecalis* in the high-risk group^27^. Esp is a factor involved in biofilm formation and contributes to the development of endocarditis and urinary tract infections^25^. We demonstrated that *E. faecalis* YM0831 did not retain the gene cluster encoding CylL, CylS, CylM, CylA, CylB, and Esp (Fig. 4b). These results also support the notion that *E. faecalis* YM0831 is a non-pathogenic strain.

### Effect of *E. faecalis* YM0831 on human hyperglycemia

**Fig. 5:**
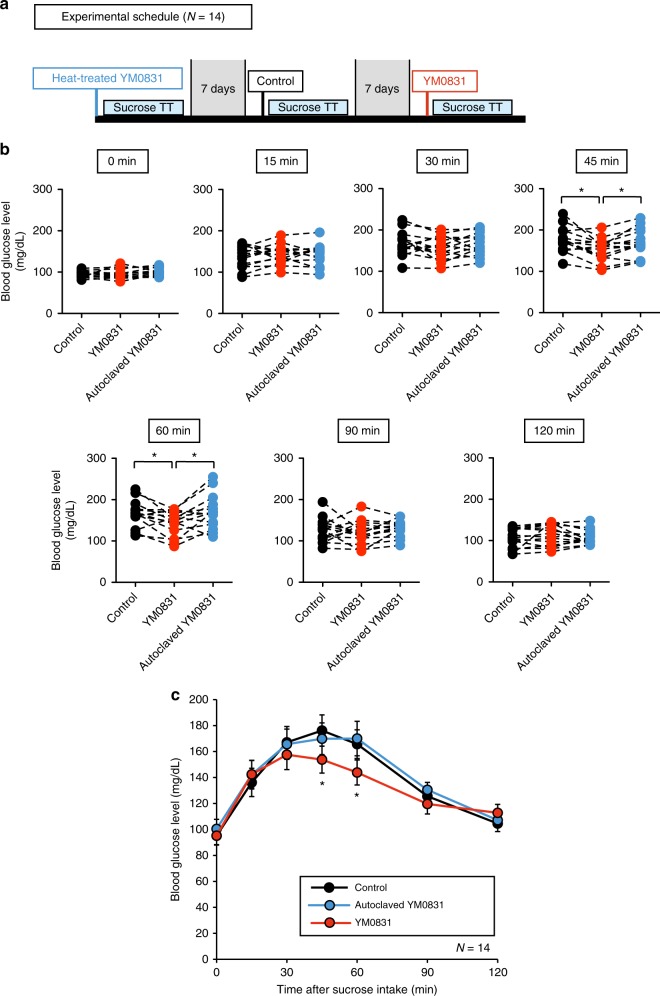
Inhibition of blood glucose increases by ingestion of *E. faecalis* YM0831 in a sucrose tolerance test in humans. Three sucrose tolerance tests were performed in each of the 14 healthy human subjects; a non-ingestion control, ingestion of bacterial cell suspension, and ingestion of heat-treated bacterial cell suspension. Subjects took the sample solution (YM0831 bacterial cells: 4 × 10^10^ cells/50 ml) suspended in saline 15 min before sucrose loading. Subsequently, the subjects drank 150 ml of 50% (w/v) sucrose solution. Blood glucose levels of the subjects were determined at 0, 15, 30, 45, 60, 90, and 120 min after sucrose challenge. Blood was collected from the fingertip and the blood sugar level was measured using a simple blood glucose meter. **a** Experimental schedule is shown. **b** Blood glucose levels in *E. faecalis* YM0831 cell suspension ingestion group (YM0831), autoclaved cell suspension ingestion group (autoclaved YM0831), and non-ingestion group (control) for each subject after sucrose loading are shown. *P < 0.017 after Bonferroni correction (paired Student’s *t-*test). **c** Sucrose tolerance tests in *E. faecalis* YM0831 cell suspension ingestion group (YM0831), autoclaved cell suspension ingestion group (Autoclaved YM0831), and non-ingestion group (control) are shown. *P < 0.017 after Bonferroni correction (paired Student’s *t-*test). Data represent mean ± SEM

We next performed human clinical trials to examine whether *E. faecalis* YM0831 exhibited inhibitory effects against an increase in blood glucose after sucrose intake. Three sucrose tolerance tests were performed in each of the 14 healthy human subjects with either no ingestion (control), or ingestion of a cell suspension of the *E. faecalis* YM0831 or autoclaved cell suspension of *E. faecalis* YM0831 (Fig. 5a and Supplementary Table 4). Blood glucose levels of the subjects were determined at 0, 15, 30, 45, 60, 90, and 120 min after the sucrose challenge. The blood glucose levels at 45 and 60 min after sucrose loading were significantly lower in the *E. faecalis* YM0831-ingesting group than in the non-ingesting control group (Fig. 5b, c). The suppressive effect by *E. faecalis* YM0831 on the increase in blood glucose after sucrose loading was abolished by autoclaving the *E. faecalis* YM0831 cells (Fig. 5b, c). These findings indicate that *E. faecalis* YM0831 has an inhibitory effect on increases in blood glucose levels after ingestion of sucrose in humans, and that the hypoglycemic factor in *E. faecalis* YM0831 is heat-sensitive.

We next performed clinical trials to examine whether the inhibitory effect of *E. faecalis* YM0831 was retained 1 day after feeding. Three sucrose tolerance tests were performed in each of the 12 healthy human subjects to whom we provided the cells of *E. faecalis* YM0831 (bacteria and heat-treated bacteria, respectively) or no ingestion (control) (Supplementary Figure 6a and Supplementary Table 4). The blood glucose levels after sucrose loading were not lower in the *E. faecalis* YM0831-ingesting group than in the non-ingesting control or heat-treated cell-ingesting groups (Supplementary Figure 6b). This result suggests that the inhibitory effect of *E. faecalis* YM0831 on increases in blood glucose induced by sucrose intake in humans was not retained 1 day after loading the bacteria.

**Fig. 6:**
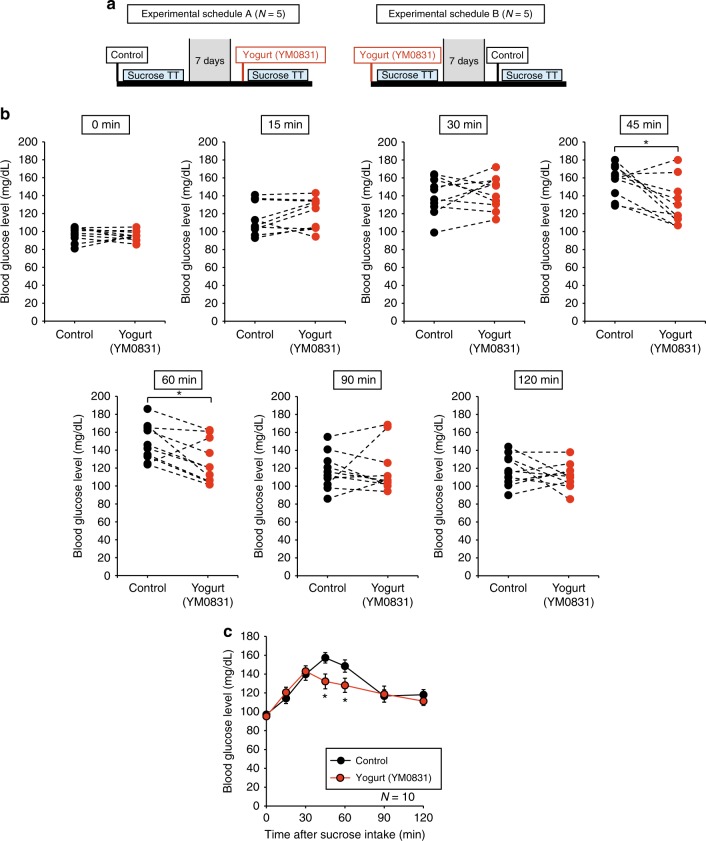
Inhibition by ingestion of a yogurt produced by *E. faecalis* YM0831 against blood glucose increases in a sucrose tolerance test in humans. Sucrose tolerance tests were conducted on 10 healthy human subjects by the crossover method with or without yogurt ingestion. The subjects consumed the yogurt sample (200 ml, YM0831 bacterial cells: 4 × 10^10^ cells/200 ml yogurt) 10 min before sucrose loading. Subsequently, the subjects drank 150 ml of 50% (w/v) sucrose solution. Blood glucose levels of the subjects were determined at 0, 15, 30, 45, 60, 90, and 120 min after sucrose challenge. Blood was collected from the fingertip and the blood sugar level was measured by using a simple blood glucose meter. **a** Experimental schedule is shown. **b** Blood glucose levels in the yogurt-ingestion group (yogurt [YM0831]) and non-ingestion group (control) for each subject for each time after sucrose loading are shown. *P < 0.05 (paired Student’s *t-*test). **c** Sucrose tolerance tests in the yogurt-ingestion group (yogurt [YM0831]) and non-ingestion group (control) are shown. *P < 0.05 (paired Student’s *t-*test). Data represent mean ± SEM

We also examined whether yogurt produced by *E. faecalis* YM0831 suppressed the increase in blood glucose after sucrose loading in humans. The two ingestion schedules shown in Fig. 6a were carried out in a total of 10 healthy humans (Supplementary Table 4). The results demonstrated that the blood glucose levels at 45 and 60 min after sucrose loading were significantly lower in the yogurt-ingesting group than in the non-ingesting control group (Fig. 6b, c). Therefore, yogurt produced by the *E. faecalis* YM0831 exhibited an inhibitory effect on increases in blood glucose levels after sucrose ingestion in humans.

## Discussion

The experiments described in the present paper revealed that lactic acid bacterium, the *E. faecalis* YM0831, which inhibits glucose uptake by Caco-2 cells, suppresses sucrose-induced hyperglycemia in healthy humans. Fermented foods such as yogurt produced by the *E. faecalis* YM0831 can also be expected to suppress postprandial hyperglycemia in humans.

Suppression by the *E. faecalis* YM0831 of an increase in blood glucose after glucose intake was also observed in the silkworm screening system. This finding suggests that the *E. faecalis* YM0831 has the potential to inhibit intestinal tract glucose transport in silkworms. Furthermore, the *E. faecalis* YM0831 suppresses glucose uptake by Caco-2 cells, which are human intestinal-derived cells. In the human intestinal tract, glucose is taken up via the glucose transporters GLUT2, SGLT-1, and SGLT-2^28,29^. Caco-2 cells express those glucose transporters^30–32^. Therefore, *E. faecalis* YM0831 might inhibit the activity of glucose transporters, such as GLUT2, SGLT-1, and SGLT-2 in the intestinal cells, thereby suppressing an increase in blood glucose induced by sucrose intake.

Useful lactic acid bacteria have been isolated from human feces. We therefore attempted to collect lactic acid bacteria from the intestinal tract of arthropods considering the possibility that animals have useful lactic acid bacteria in their intestinal tract, and successfully isolated *E. faecalis* YM0831 as a functional lactic acid bacterium from the intestinal tract of a chilopod.

We isolated a transposon insertion-mutant that reduced the inhibitory activity of *E. faecalis* YM0831 against glucose uptake by Caco-2 cells, and found that the mutant also had decreased inhibitory activity against sucrose-induced hyperglycemia in silkworms. The findings suggest that the inhibitory activity of *E. faecalis* YM0831 against glucose uptake by Caco-2 cells directly contributes to suppress sucrose-induced hyperglycemia in host animals.

The *man* operon, which is necessary for the inhibitory activity against glucose uptake by Caco-2 cells, contains genes encoding components of the mannose phosphotransferase system (man-PTS), including an enzyme that synthesizes mannose-6-phosphate. Bacterial man-PTS plays a role in converting mannose or glucose outside the cell into mannose-6-phosphate or glucose 6-phosphate, and transporting it into the cell^33^. In Gram-negative bacteria, mannose-6-phosphate becomes GDP-mannose by phosphomannose isomerase/GDP-d-mannose pyrophosphorylases^33^. GDP-d-mannose is a substrate for the mannosylation reaction involved in the biosynthesis of lipopolysaccharides^33^. On the other hand, because *E. faecalis*, a Gram-positive bacterium, does not have genes encoding the enzymes that synthesize GDP-mannose from mannose-6-phosphate reported in Gram-negative bacteria, it is unclear how mannose-6-phosphate is used in *E. faecalis*. In *E. faecalis*, man-PTS is involved in regulating the expression of various genes, including those involved in sugar metabolism^34,35^. We therefore assumed that the general gene expression regulated by the mannose operon and man-PTS might be involved in the inhibitory action of *E. faecalis* YM0831.

We found that the soluble substance produced by *E. faecalis* YM0831 inhibits glucose uptake in Caco-2 cells. We therefore assume that the substance produced by *E. faecalis* YM0831 inhibits glucose uptake in the intestinal tract, and that this inhibition leads to suppression of the blood glucose increase following the ingestion of sucrose. We hypothesized that this soluble substance is synthesized in *E. faecalis* YM0831 in a man-PTS-dependent manner. Identification of the active substances is an important subject for a future study.

As shown in Fig. 3c, d, there were statistically significant differences between no bacteria and Tp10-72, which is a transposon mutant derived from *E. faecalis* YM0831DR. Figure 3b shows that there was no significant difference between no bacteria and Tp10-72 in Caco-2 cells. These results suggest that the inhibitory activities against sucrose-induced or glucose-induced hyperglycemia in silkworms remains in Tp10-72. Moreover, autoclaving *E. faecalis* YM0831 decreased its suppressive effect on sucrose-induced hyperglycemia in silkworms, but when the dose of the autoclaved *E. faecalis* YM0831 in the diet was increased to 25% [w/w], the suppressive effect was still observed (Supplementary Figure 7). This finding suggests that active substances in *E. faecalis* YM0831 that are resistant to autoclave treatment suppress sucrose-induced hyperglycemia. Identification of the active substances from Tp10-72 and the autoclaved *E. faecalis* YM0831 is the subject of a future study.

*E. faecalis* YM0831 does not have pathogenesis-related genes encoding cytolysin and Esp, and this lactic acid bacterial strain is not classified in the high-risk enterococci group. Non-pathogenic *E. faecalis* strains, such as Symbioflor, are traditionally used to produce fermented foods^23,24^. Therefore, we conclude that *E. faecalis* YM0831 is a safe lactic acid bacteria strain for the production of fermented foods. Additionally, *E. faecalis* YM0831 did not show cytotoxicity against Caco-2 cells in vitro (Fig. 2f). Moreover, in human clinical trials in this study, no adverse events appeared to result from administration of *E. faecalis* YM0831. These findings support the potential use of *E. faecalis* YM0831 for preventive human consumption. A comparison of the inhibitory effect between *E. faecalis* YM0831 and other *E. faecalis* strains is the subject of a future study.

Lifestyle-related diseases such as obesity and diabetes caused by intake of a high-calorie diet containing sucrose are serious problems in today’s society. Prevention of day to day increases in blood glucose is very important to prevent the onset of lifestyle-related diseases. Recently, a phenomenon called a blood glucose level spike, i.e., a sharp rise in postprandial blood glucose induced following the intake of beverages containing a high concentration of sucrose, was recognized to be common among young people and may lead to an onset of diabetes^36^. Restriction of a sucrose-containing diet is considered to be effective for preventing the postprandial increase in blood glucose^37^. This strategy is often difficult to continually implement, however, because many people cannot resist the desire to eat high-calorie food. Food additives that suppress an increase in blood glucose level may be effective for preventing an increase in the blood glucose level. The effectiveness of lactic acid bacteria for suppressing a blood glucose increase after sucrose loading in animal models using mice has been reported^12^. No reports to date, however, have provided direct evidence that lactic acid bacteria suppress the increase in blood glucose induced by sucrose intake in humans. Here, we demonstrated the *E. faecalis* YM0831 has this capacity. Furthermore, yogurt produced by *E. faecalis* YM0831 also exhibited suppressive effects on sucrose-induced hyperglycemia in healthy humans. On the other hand, 1 day after ingestion of *E. faecalis* YM0831, hyperglycemia caused by sucrose in healthy humans was not suppressed. We assume that the number of *E. faecalis* YM0831 cells is decreased in human intestine after 1 day and the amount of the active substance is no longer sufficient. In the present study, it is not clear how long *E. faecalis* YM0831 is effective between 2 and 24 h after ingestion. Moreover, the most effective dose of *E. faecalis* YM0831 has not been established. This information is necessary for *E. faecalis* YM0831 to be considered as a supplement in functional food. To evaluate the usefulness of *E. faecalis* YM0831 for producing functional yogurt, the activity of yogurt produced by *E. faecalis* YM0831 must be compared with that of yogurt produced by other lactic acid bacteria. The blood glucose level of some humans who ingested the yogurt produced by *E. faecalis* YM0831 was high at 30 min after ingesting sucrose. This means that individual differences among humans affects the activity of the yogurt. Further studies with a large-scale and a long-term clinical trial are necessary to investigate whether yogurt produced by *E. faecalis* YM0831 is effective for preventing diabetes onset. In addition, the efficacy of yogurt produced by *E. faecalis* YM0831 against type II diabetes patients is an important subject for future studies.

In conclusion, our results indicate that a lactic acid bacteria strain, *E. faecalis* YM0831, suppresses sucrose-induced postprandial hyperglycemia in humans and provide mechanistic insight that *E. faecalis* YM0831 inhibits glucose uptake by human intestinal epithelial cells in *man* operon expression-dependent manner. A preventive medicine approach using lactic acid bacteria might thus be effective against the onset of diabetes.

## Methods

### Culture of lactic acid bacteria

Lactic acid bacteria isolated from various sources were grown in de Man, Rogosa, Sharpe (MRS: Becton Dickinson, Franklin Lakes, NJ, USA) medium containing 0.5% calcium carbonate at 30 °C for 1–3 days under aerobic conditions. The lactic acid bacteria were grown in MRS liquid medium at 30 °C for 1–3 days under static conditions.

### Measurement of glucose levels in hemolymph of silkworms

Silkworms were reared according to the previously reported method^17,38,39^. Fifth-instar larvae were used in this study. A diet containing 10% (w/w) sucrose or glucose was prepared by mixing an artificial diet (Silkmate 2S: Nihon Nosan Co., Ltd., Kanagawa, Japan) and d-sucrose or d-glucose. A diet containing lactic acid bacteria was prepared by mixing the diet containing 10% sucrose or glucose and a pellet of lactic acid bacteria harvested by centrifugation of a full-growth culture. Silkworms were fed the diet containing sugar and lactic acid bacteria at 27 °C for 1 h. We used 0.9–1.1 g of silkworms and fed 0.7 g per larvae. Each silkworm eats ~0.2 g of food after 1 h^17^. Hemolymph was collected by cutting the first proleg, and glucose levels in the hemolymph were determined using a glucometer (Accu-Chek: Roche, Basel, Switzerland)^17^.

### Identification of lactic acid bacteria

Lactic acid bacteria were Gram stained. Genes encoding rRNA were sequenced using previously reported primers (9F:GAGTTTGATCCTGGCTCAG and 1541R:AAGGAGGTGATCCAGCC)^17^. The PCR reaction mixture was incubated as 94 °C for 2 min and then for 30 cycles (94 °C, 15 s; 55 °C, 30 s; 68 °C 2 min). Bacterial species were identified based on ≥99% sequence matching using NCBI BLAST (https://blast.ncbi.nlm.nih.gov/Blast.cgi). The rRNA sequence has been deposited in Genbank (accession number; MK182799). The *E. faecalis* YM0831 (0831-07) has been deposited in National Institute of Technology and Evaluation (Deposit number: P-02309, NITE, Tokyo, Japan).

### Glucose transport assay using silkworm intestine

Fifth-instar larvae of silkworms were anesthetized by being placed on ice for 10 min. The heads of the silkworms were cut off with scissors, and the digested food was removed. Glucose solution (100 mg/ml) was enclosed in isolated silkworm intestinal tract. The intestinal samples were incubated in PBS at 27 °C. Glucose levels outside of the intestine were determined using a glucometer (Accu-Chek: Roche).

### Glucose uptake assay

Glucose uptake by Caco-2 cells was determined by the method described previously^18^. Caco-2 cells were obtained from American Type Cell Collection (ATCC, Manassas, VA, USA) and cultured in DMEM (Gibco, NY, USA) containing 10% FBS (Gibco), 1% penicillin/streptomycin (Gibco) at 37 °C with 5% CO_2_ in air. Caco-2 cells were cultured in a monolayer in a 96-well plate (Tissue culture testplate 96F: TPP, Switzerland). The cells were incubated for an additional 24 h in the serum-free DMEM. Subsequently, cells were washed with Na buffer [10 mM HEPES (pH 7.4), 140 mM NaCl, 20 mg/ml BSA] and incubated in Na buffer for 15 min. After incubation, cells were incubated in Na buffer with 50 µM 2-deoxy-2-[(7-nitro-2, 1, 3-benzoxadiazol-4-yl)amino]-d-glucose (2-NBDG: Cayman Chemical, MI, USA) for 10 min and then washed with ice-cold PBS twice to remove the 2-NBDG. The fluorescence of cells containing 2-NBDG was detected by fluorescence microscopy (IX73: Olympus, Tokyo, Japan) and calculated by Image J ver. 1.43u (National Institutes of Health, USA).

Caco-2 cells were cultured in a monolayer in a 96-well plate. After removal of the culture medium by aspiration, Na buffer [10 mM HEPES (pH 7.4), 140 mM NaCl, 20 mg/ml BSA] was added, and cells were further incubated at 37 °C for 10 min. After washing the cells twice with 200 μl of PBS, 100 μl of water-soluble tetrazolium salt-1 (WST-1) solution (Roche, Basel, Switzerland) diluted 1/10 with PBS was added and samples were incubated at 37 °C for 1 h. The OD_450_ values were measured using a microplate reader (iMark: BioRad, CA, USA).

### Transposon mutagenesis

*E. faecalis* YM0831 was subjected to ethylmethane sulfonate-induced mutagenesis to obtain YM0831DR, which was resistant to both rifampicin and kanamycin. The ethylmethane sulfonate treatment and selection of drug-resistant strains followed previously reported methods^40^. YM0831DR can grow in Todd–Hewitt broth (THB: Becton Dickinson, MD, USA) with kanamycin (final conc. 25 µg/ml) and with rifampicin (final conc. 25 µg/ml). Mutagenesis was carried out by a filter mating method as described previously, with some modifications^41^. The donor, *E. faecalis* JH2SS/pAM378, and the recipient, *E. faecalis* YM0831DR, were grown without shaking at 37 °C overnight in THB with tetracycline (final conc. 25 µg/ml), and with rifampicin (final conc. 25 µg/ml) and kanamycin (final conc. 25 µg/ml), respectively. Five-milliliter cultures of each strain were mixed and collected on a membrane filter (filter type; 0.45 µm HV: Millipore, BTV, USA). The filters were placed on THB agar (THB with 1.5% agar) containing 4% horse blood (Nippon Bio-test, Saitama, Japan) and incubated at 37 °C for 18 h. The bacteria were then suspended in 2 ml THB and spread on THB agar plates containing tetracycline (final conc. 25 µg/ml), rifampicin (final conc. 25 µg/ml), and kanamycin (final conc. 25 µg/ml). After overnight incubation at 37 °C, 1026 independent colonies were picked and used as a transposon mutant library obtained from *E. faecalis* YM0831DR.

### Quantitative reverse transcription (RT)-PCR analysis

Parent strain (YM0831DR) and Tp10-72 were cultured in MRS liquid medium for 2 days, and the cells were harvested by centrifugation. Total RNA from YM0831DR and Tp10-72 was extracted using an RNeasy Mini kit (Qiagen, Hilden, Germany), according to the manufacturer’s protocol. RT-PCR was carried out according to the previously reported method^42^. Contaminated genomic DNA in the total RNA samples was digested by RQ1 RNase-free DNase (Promega, WI, USA). The RNA was reverse-transcribed to cDNA using TaqMan RT reagents (Applied Biosystems, CA, USA). The primers used in this study are shown in Supplementary Table 5. Quantitative real-time polymerase chain reaction was performed using FastStart SYBR Green Master (Roche), according to the manufacturer’s protocol.

### Plasmid construction

Plasmid construction was performed using the In-Fusion HD Cloning kit (Takara Bio USA, CA, USA), according to the manufacturer’s protocol. The *man* operon was amplified with PCR from *E. faecalis* YM0831 chromosomal DNA using the primers *man* operon-F and *man* operon-R. The pND50 fragment was amplified with PCR from pND50 vector^43^ using the primers pND50infusion-F and pND50infusion-R. The primers used in this study are shown in Supplementary Table 5. Plasmid (pMan operon) have been deposited in Addgene (Deposit number; 76594).

### Comparative genome analysis

Chromosomal DNA of *E. faecalis* YM0831 was extracted using a QIA blood DNA kit (Qiagen, Hilden, Germany). The whole genome sequence of the *E. faecalis* YM0831 was determined by Ion-PGM (Thermo Fisher Scientific, Franklin, MA, USA) and analyzed using a CLC genomic workbench (Qiagen). Sequence typing was performed based on the sequences of the *zwf*, *gap-2*, *pstS*, *glcK*, *aroE*, *xpt*, and *ygiL* genes using eBURST V3 (eburst.mlst.net/V3/mlst_datasets/). The gene sequences of each strain in CC2, CC4, CC9, CC21, CC25, CC28, and CC40 were arranged in Clustal Omega (www.ebi.ac.uk/Tools/msa/clustalo/) and the phylogenetic tree of each strain was determined by analysis using Simple Phylogeny (www.ebi.ac.uk./Tools/services/web_simple_phylogeny/toolform.ebi). Using the sequence of the pathogenic island of MMH 594 as a template, comparative genomic analysis of the sequence of the pathogenic island was performed from the whole genome data of the *E. faecalis* YM0831 using the CLC genomic workbench. This Whole Genome Shotgun project has been deposited at DDBJ/ENA/GenBank under the accession SJRZ00000000. The version described in this paper is version DDBJ/ENA/GenBank under the accession SJRZ01000000.

### Human clinical study

Suppressive effects on increases in blood glucose in humans by *E. faecalis* YM0831 were investigated using a sucrose tolerance test. Blood glucose levels of the subjects were determined at 0, 15, 30, 45, 60, 90, and 120 min after a sucrose challenge. Blood was collected from the fingertip and the blood sugar level was measured using a simple blood glucose meter. The objectives of the present study were to evaluate whether the *E. faecalis* YM0831 inhibited the increase in the blood glucose level after sucrose intake (*n* = 14), whether the inhibitory effect of *E. faecalis* YM0831 remained 1 day after feeding (*n* = 12), and whether yogurt produced by *E. faecalis* YM0831 (*n* = 10) suppressed the increase in blood glucose after sucrose loading in healthy adult subjects. The human clinical studies were conducted at Osaki Hospital Tokyo Heart Center, Tokyo, Japan. The studies were approved by the Institutional Ethics Committee of the study site and carried out in accordance with Japan Ethical Guidelines for Medical and Health Research Involving Human Subjects. All subjects provided written informed consent before initiation of any study procedures. These studies were registered at the Japan UMIN Clinical Trials Registry (UMIN000024338 for Fig. 5 and Supplementary Figure 6, and UMIN000020807 for Fig. 6).

### Statistics

The data are shown as the mean ± SEM. Unless otherwise noted, the significance of differences was calculated using a two-tailed Student’s *t*-test at the significance level *P* < 0.05.

### Reporting summary

Further information on research design is available in the Nature Research Reporting Summary linked to this article.

## Supplementary information

Supplementary Information

Description of Additional Supplementary Files

Reporting Summary

Supplementary Data 1

## Data Availability

All data generated or analyzed during this study are included in this published Article and its Supplementary files. Raw data used to generate the figures in this manuscript are included in Supplementary Data 1. The GenBank accession number for the rRNA nucleotide sequence is MK182799. The whole genome shotgun project has been deposited at DDBJ/ENA/GenBank: SUBID = SUB5246687, BioProject = PRJNA524752, BioSample = SAMN11032480, Accession number = SJRZ00000000, and Organism = *Enterococcus faecalis* YM083. The *E. faecalis* YM0831 (0831-07) has been deposited in National Institute of Technology and Evaluation (Deposit number: P-02309, NITE, Tokyo, Japan). Plasmid (pMan operon) have been deposited in Addgene (Deposit number; 76594).

## References

[1] Johnson RJ (2013). Sugar, uric acid, and the etiology of diabetes and obesity. Diabetes.

[2] Ochoa M, Lalles JP, Malbert CH, Val-Laillet D (2015). Dietary sugars: their detection by the gut-brain axis and their peripheral and central effects in health and diseases. Eur. J. Nutr..

[3] Zimmet P, Alberti KG, Shaw J (2001). Global and societal implications of the diabetes epidemic. Nature.

[4] Newens K. J., Walton J. (2015). A review of sugar consumption from nationally representative dietary surveys across the world. Journal of Human Nutrition and Dietetics.

[5] Pereira MA (2014). Sugar-sweetened and artificially-sweetened beverages in relation to obesity risk. Adv. Nutr..

[6] Lewis AS (2013). Comparison of 5% versus 15% sucrose intakes as part of a eucaloric diet in overweight and obese subjects: effects on insulin sensitivity, glucose metabolism, vascular compliance, body composition and lipid profile. A randomised controlled trial. Metabolism.

[7] Reiser S, Bickard MC, Hallfrisch J, Michaelis OEt, Prather ES (1981). Blood lipids and their distribution in lipoproteins in hyperinsulinemic subjects fed three different levels of sucrose. J. Nutr..

[8] Black RN (2006). Effect of eucaloric high- and low-sucrose diets with identical macronutrient profile on insulin resistance and vascular risk: a randomized controlled trial. Diabetes.

[9] Bischoff H (1994). Pharmacology of alpha-glucosidase inhibition. Eur. J. Clin. Invest..

[10] Xiao JB, Hogger P (2015). Dietary polyphenols and type 2 diabetes: current insights and future perspectives. Curr. Med. Chem..

[11] McCranie EK, Bachmann BO (2014). Bioactive oligosaccharide natural products. Nat. Prod. Rep..

[12] Honda K, Moto M, Uchida N, He F, Hashizume N (2012). Anti-diabetic effects of lactic acid bacteria in normal and type 2 diabetic mice. J. Clin. Biochem. Nutr..

[13] Panwar H, Calderwood D, Grant IR, Grover S, Green BD (2014). *Lactobacillus* strains isolated from infant faeces possess potent inhibitory activity against intestinal alpha- and beta-glucosidases suggesting anti-diabetic potential. Eur. J. Nutr..

[14] Matsumoto Y (2015). Diabetic silkworms for evaluation of therapeutically effective drugs against type II diabetes. Sci. Rep..

[15] Matsumoto Y, Sumiya E, Sugita T, Sekimizu K (2011). An invertebrate hyperglycemic model for the identification of anti-diabetic drugs. PLoS One.

[16] Matsumoto Y (2014). Transgenic silkworms expressing human insulin receptors for evaluation of therapeutically active insulin receptor agonists. Biochem. Biophys. Res. Commun..

[17] Matsumoto Y, Ishii M, Sekimizu K (2016). An *in vivo* invertebrate evaluation system for identifying substances that suppress sucrose-induced postprandial hyperglycemia. Sci. Rep..

[18] Yamabe N, Kang KS, Lee W, Kim SN, Zhu BT (2015). Estriol blunts postprandial blood glucose rise in male rats through regulating intestinal glucose transporters. Am. J. Physiol. Endocrinol. Metab..

[19] Goh H. M. Sharon, Yong M. H. Adeline, Chong Kelvin Kian Long, Kline Kimberly A. (2017). Model systems for the study of Enterococcal colonization and infection. Virulence.

[20] Flores-Mireles AL, Walker JN, Caparon M, Hultgren SJ (2015). Urinary tract infections: epidemiology, mechanisms of infection and treatment options. Nat. Rev. Microbiol..

[21] Ruiz-Garbajosa P (2006). Multilocus sequence typing scheme for *Enterococcus faecalis* reveals hospital-adapted genetic complexes in a background of high rates of recombination. J. Clin. Microbiol..

[22] Solheim M (2011). Comparative genomic analysis reveals significant enrichment of mobile genetic elements and genes encoding surface structure-proteins in hospital-associated clonal complex 2 *Enterococcus faecalis*. BMC Microbiol..

[23] Fritzenwanker, M. et al. Complete genome sequence of the probiotic *Enterococcus faecalis* symbioflor 1 clone DSM 16431. *Genome Announc.* **1**10.1128/genomeA.00165-12 (2013). 10.1128/genomeA.00165-12 PMC3569346 23405346

[24] Martens U, Enck P, Zieseniss E (2010). Probiotic treatment of irritable bowel syndrome in children. Ger. Med. Sci..

[25] Arias CA, Murray BE (2012). The rise of the *Enterococcus*: beyond vancomycin resistance. Nat. Rev. Microbiol..

[26] McBride SM (2009). Genetic variation and evolution of the pathogenicity island of *Enterococcus faecalis*. J. Bacteriol..

[27] Coburn PS, Gilmore MS (2003). The *Enterococcus faecalis* cytolysin: a novel toxin active against eukaryotic and prokaryotic cells. Cell. Microbiol..

[28] Chen Lihong, Tuo Biguang, Dong Hui (2016). Regulation of Intestinal Glucose Absorption by Ion Channels and Transporters. Nutrients.

[29] Mudaliar S, Polidori D, Zambrowicz B, Henry RR (2015). Sodium-glucose cotransporter inhibitors: effects on renal and intestinal glucose transport: from bench to bedside. Diabetes Care.

[30] Johnston K, Sharp P, Clifford M, Morgan L (2005). Dietary polyphenols decrease glucose uptake by human intestinal Caco-2 cells. FEBS Lett..

[31] Mahraoui L (1994). Presence and differential expression of SGLT1, GLUT1, GLUT2, GLUT3 and GLUT5 hexose-transporter mRNAs in Caco-2 cell clones in relation to cell growth and glucose consumption. Biochem. J..

[32] Mesonero J (1994). Expression of the hexose transporters GLUT1-GLUT5 and SGLT1 in clones of Caco-2 cells. Biochem. Soc. Trans..

[33] Wu B, Zhang Y, Zheng R, Guo C, Wang PG (2002). Bifunctional phosphomannose isomerase/GDP-D-mannose pyrophosphorylase is the point of control for GDP-D-mannose biosynthesis in Helicobacter pylori. FEBS Lett..

[34] Opsata M, Nes IF, Holo H (2010). Class IIa bacteriocin resistance in *Enterococcus faecalis* V583: the mannose PTS operon mediates global transcriptional responses. BMC Microbiol..

[35] Suarez C, Espariz M, Blancato VS, Magni C (2013). Expression of the agmatine deiminase pathway in *Enterococcus faecalis* is activated by the AguR regulator and repressed by CcpA and PTS(Man) systems. PLoS One.

[36] Sottero B (2015). Postprandial dysmetabolism and oxidative stress in Type 2 diabetes: pathogenetic mechanisms and therapeutic strategies. Med. Res. Rev..

[37] Snorgaard O, Poulsen GM, Andersen HK, Astrup A (2017). Systematic review and meta-analysis of dietary carbohydrate restriction in patients with type 2 diabetes. BMJ Open Diabetes Res. Care.

[38] Kaito C, Akimitsu N, Watanabe H, Sekimizu K (2002). Silkworm larvae as an animal model of bacterial infection pathogenic to humans. Microb. Pathog..

[39] Kurokawa K, Kaito C, Sekimizu K (2007). Two-component signaling in the virulence of *Staphylococcus aureus*: a silkworm larvae-pathogenic agent infection model of virulence. Methods Enzymol..

[40] Matsumoto Y (2016). A critical role of mevalonate for peptidoglycan synthesis in *Staphylococcus aureus*. Sci. Rep..

[41] Maki H, Yamaguchi T, Murakami K (1994). Cloning and characterization of a gene affecting the methicillin resistance level and the autolysis rate in *Staphylococcus aureus*. J. Bacteriol..

[42] Miyashita A, Takahashi S, Ishii K, Sekimizu K, Kaito C (2015). Primed immune responses triggered by ingested bacteria lead to systemic infection tolerance in silkworms. PLoS One.

[43] Kaito C (2005). Silkworm pathogenic bacteria infection model for identification of novel virulence genes. Mol. Microbiol..

