# Supplementary material for: Enterococcus faecalis YM0831 suppresses sucrose-induced hyperglycemia in a silkworm model and in humans
Source: Commun Biol. 2019 May 2;2:157. doi: 10.1038/s42003-019-0407-5 (PMC6497652; doi:10.1038/s42003-019-0407-5)
Supplement: Supplementary file 1 — Supplementary Information [file 42003_2019_407_MOESM1_ESM.pdf]

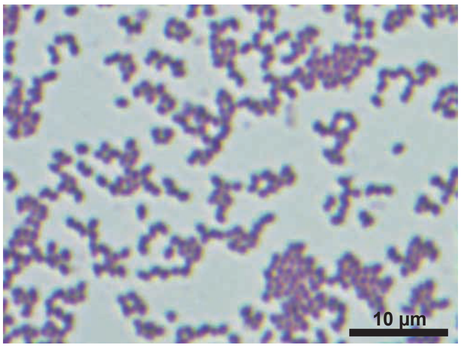

Supplementary Figure 1 | Gram staining of *E. faecalis* YM0831.

E. faecalis YM0831 was grown in MRS liquid medium at 30°C for 1 day under static condition. Gram staining was performed using Gram-color (Merck, Darmstadt, Germany), according to the manufacturer's protocol.

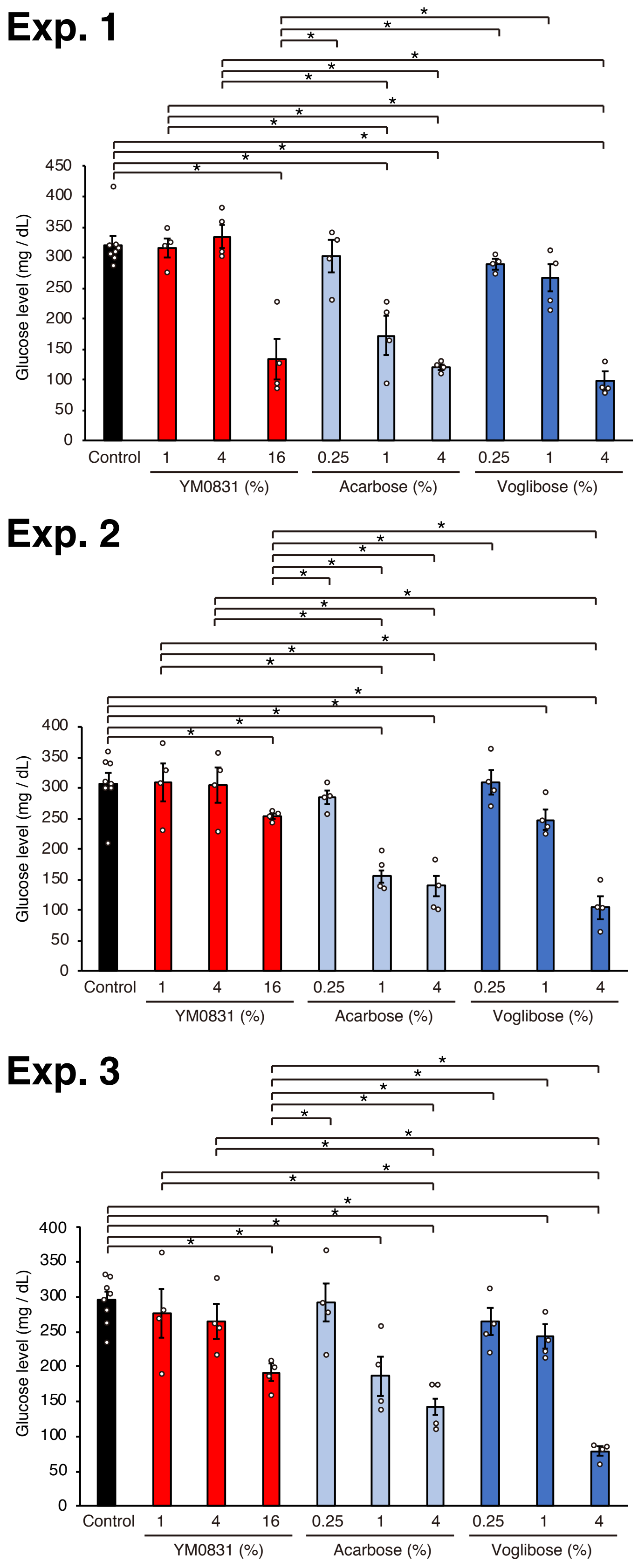

Exp. 1
Glucose level (mg / dL)
Control
1
4
16
0.25
1
4
0.25
1
4
YM0831 (%)
Acarbose (%)
Voglibose (%)
Exp. 2
Exp. 3

Supplementary Figure 2 | Effects of *E. faecalis* YM0831, acarbose, and voglibose against an increase in silkworm hemolymph glucose levels induced by sucrose intake.

Silkworms were fed a diet containing 10% (w/w) sucrose with or without *E. faecalis* YM0831 (1, 4, 16% [w/w] in the diet), acarbose (0.25, 1, 4% [w/w] in the diet), and voglibose (0.25, 1, 4,% [w/w] in the diet) for 1 h. Glucose levels in the silkworm hemolymph were measured (n = 4-8/group). Data represent mean ± SEM. Statistically significant differences between groups were evaluated using Student's *t*-test. * : $P < 0.05$. The results of three independent experiments (Exp. 1, Exp. 2, and Exp. 3) were shown.

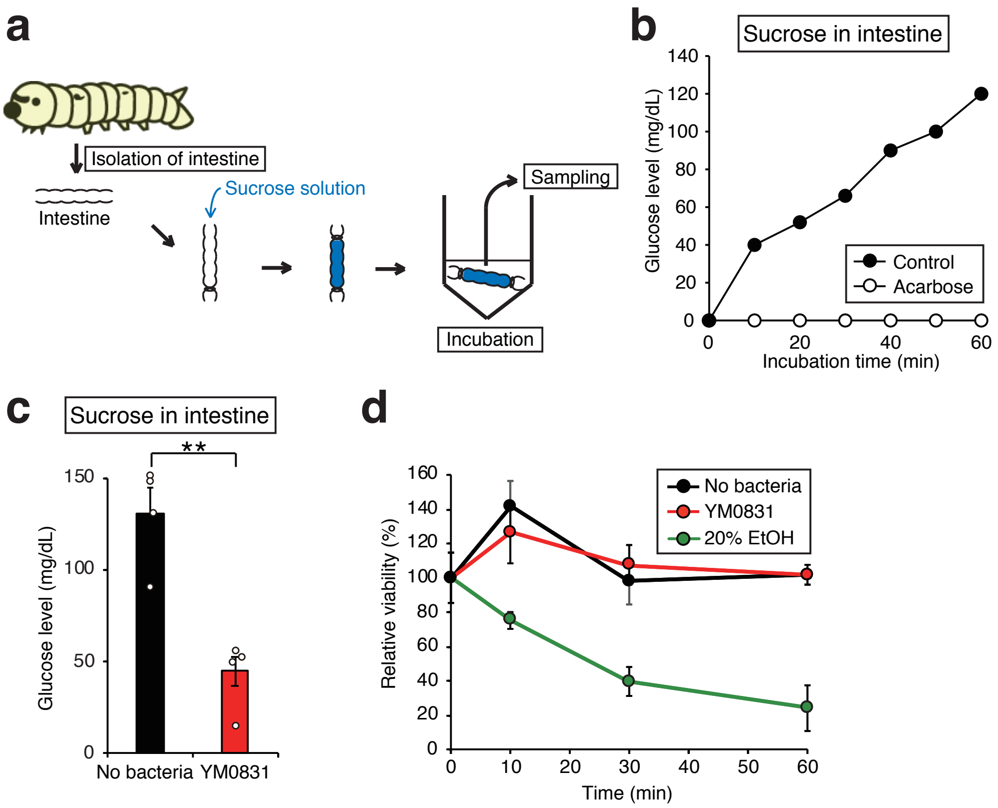

Supplementary Figure 3 | Inhibitory effect of the addition of acarbose on the transport of sucrose in isolated silkworm intestine.

(**a**) Schematic illustration of the *in vitro* sugar transport assay using isolated silkworm intestine. (**b**) Sucrose solution (100 mg/ml) or sucrose solution containing acarbose (40 mg/ml) were enclosed in isolated silkworm intestine. The intestinal samples were incubated in PBS at 27°C. Glucose levels outside of the intestine were determined. (**c**) Sucrose solution (100 mg/ml) with or without *E. faecalis* YM0831 (YM0831, 250 mg wet weight/ml) was enclosed in isolated silkworm intestinal tract. Intestinal samples were incubated in PBS at 27°C for 60 min. Glucose levels outside of the intestine were determined. Data represent mean ± SEM. Statistically significant differences between groups were evaluated using Student's *t*-test. ** : $P < 0.01$. n = 4/group. (**d**) Viability of isolated intestine following the addition or absence of *E. faecalis* YM0831 (250 mg wet weight/ml) or addition of 20% ethanol solution was measured. Data represent mean ± SEM. n = 3/group.

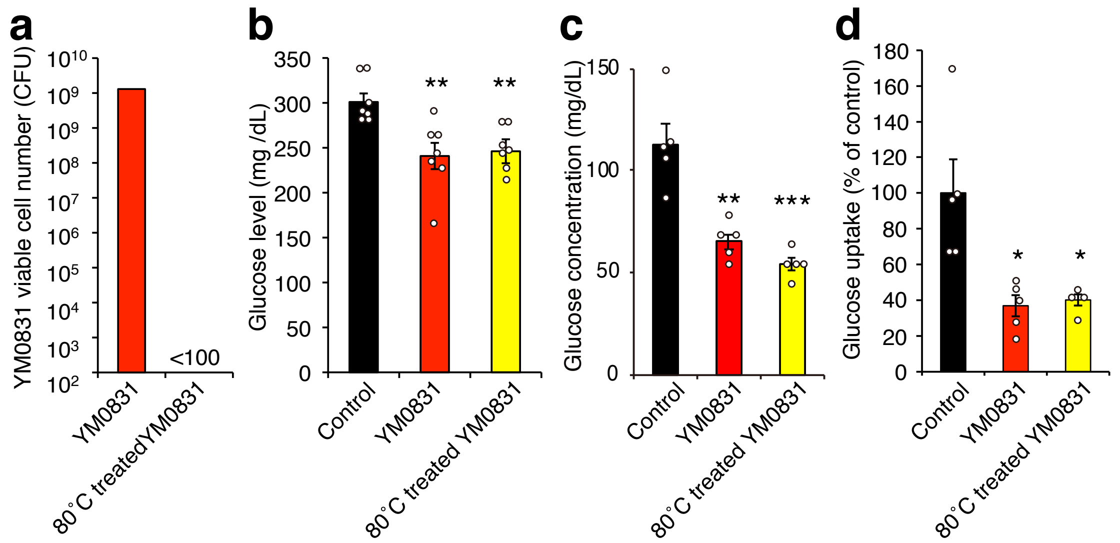

Supplementary Figure 4 | Inhibitory effect of the 80˚C treated *E. faecalis* YM0831 against an increase in hemolymph glucose levels in silkworms induced by intake of sucrose.

(**a**) Viable cell number was determined by colony count method using agar plate. The sample solutions of non-treated *E. faecalis* YM0831 (YM0831) or *E. faecalis* YM0831 treated at 80˚C for 15 min (80˚C treated YM0831) were serially diluted and the diluted samples were spread on MRS agar plate. The plates were incubated at 37˚C overnight. Colony number on the plates was counted. (**b**) Silkworms were fed a diet containing 10% (w/w) sucrose with or without *E. faecalis* YM0831 (YM0831, 12.5% [w/w] in diet) or *E. faecalis* YM0831 treated at 80˚C for 15 min (80˚C treated YM0831, 12.5% [w/w] in the diet) for 1 h. Glucose levels in the silkworm hemolymph were measured (n = 7/group). Data represent mean ± SEM. Statistically significant differences between groups were evaluated using Student's *t*-test. ** : $P < 0.01$. (**c**) Glucose solution with or without *E. faecalis* YM0831 (YM0831, 250 mg wet weight/ml), or glucose solution with *E. faecalis* YM0831 treated at 80˚C for 15 min (80˚C treated YM0831) was enclosed in isolated silkworm intestinal tract. Intestinal samples were incubated in PBS at 27°C for 10 min. Glucose levels outside of the intestine were determined. Data represent mean ± SEM. Statistically significant differences between groups were evaluated using Student's *t*-test. ** : $P < 0.01$. *** : $P < 0.001$. n = 5/group. (**d**) *E. faecalis* YM0831 (YM0831, 62.5 mg wet weight cells /ml) or *E. faecalis* YM0831 treated at 80˚C for 15 min (80˚C treated YM0831, 62.5 mg wet weight cells /ml) were added in the uptake system of 2-NBDG in Caco-2 cells and fluorescence uptake in the Caco-2 cells was measured. Data represent mean ± SEM. Statistically significant differences between control and groups in the presence of samples were evaluated using Student's *t*-test. * : $P < 0.05$. n = 4-5/group.

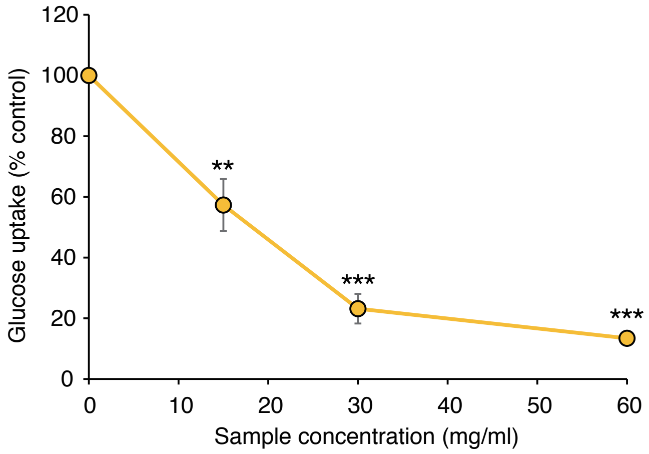

Supplementary Figure 5 | Inhibitory effect of a soluble fraction obtained by sonication of *E. faecalis* YM0831 cells on glucose uptake by Caco-2 cells.

E. faecalis YM0831 cells were suspended with water and sonicated at amplitude 30 for 5 min (Q55, QSonica, LCC., CT, USA). The sonicated sample was centrifuged and the supernatant was collected as a soluble fraction. The soluble fraction was added in the uptake system of 2-NBDG in Caco-2 cells and fluorescence uptake in the Caco-2 cells was measured. Data represent mean ± SEM. Statistically significant differences between control and groups in the presence of samples were evaluated using Student's *t*-test. ** : $P < 0.01$. *** : $P < 0.001$. n = 3-15/group.

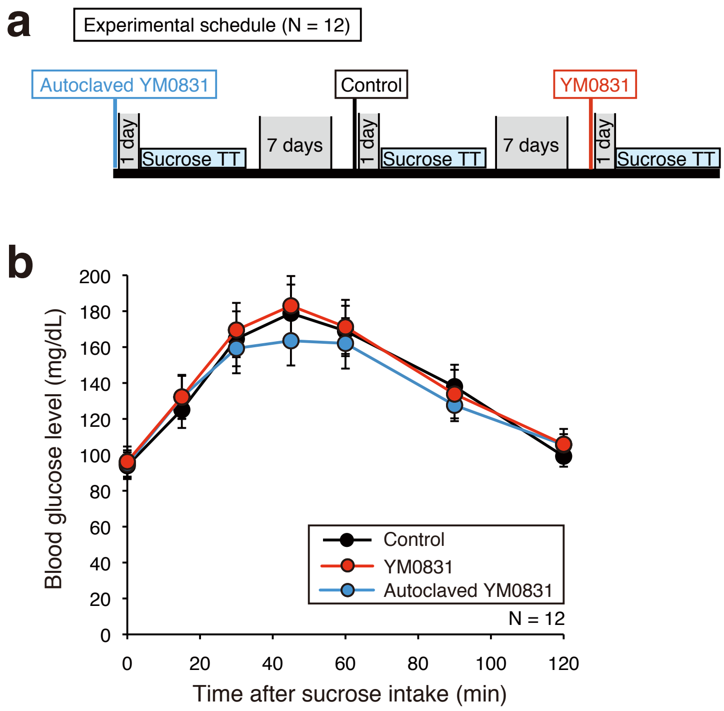

Supplementary Figure 6 | Effect in 1 day after ingestion of *E. faecalis* YM0831 against blood glucose level increases in sucrose tolerance test in humans.

Three sucrose tolerance tests were performed in each of the 12 healthy human subjects; non-ingestion control, ingestion of bacterial cell suspension, and ingestion of heat-treated bacterial cell suspension. Subjects consumed the sample solution (YM0831 bacterial cells: 4 x 10^{10} cells / 50 ml) suspended in saline 1 day before sucrose loading. Subsequently, the subjects drank 150 mL of 50% (w/v) sucrose solution. Blood glucose levels of the subjects were determined at 0, 15, 30, 45, 60, 90, and 120 min after sucrose challenge. Blood was collected from the fingertip and the blood sugar level was measured using a simple blood glucose meter. (**a**) Experimental schedule is shown. (**b**) Sucrose tolerance tests in *E. faecalis* YM0831 cell suspension ingestion group (YM0831), autoclave-treated cell suspension ingestion group (Autoclaved YM0831), and non-ingestion group (Control) are shown. Data represent mean ± SEM.

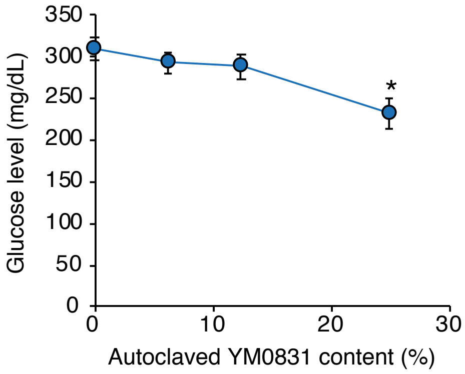

Supplementary Figure 7 | Inhibitory effect of autoclaved *E. faecalis* YM0831 against an increase in silkworm hemolymph glucose levels induced by sucrose intake.

Silkworms were fed a diet containing 10% (w/w) sucrose with or without autoclaved *E. faecalis* YM0831 (6.3, 12.5, 25% [w/w] in the diet) for 1 h. Glucose levels in the silkworm hemolymph were measured (n = 11-14/group). Data represent mean ± SEM. Statistically significant differences between groups were evaluated using Student's *t*-test. * : $P < 0.05$.

Supplementary Table 1 | Screening of lactic acid bacteria strains that suppress sucrose-induced hyperglycemia.

Strain	Species	Source	Activity[a]
#Ef-1 (YM0831)	*Enterococcus faecalis*	Chilopod	+
#Ec-1	*Enterococcus casseliflavus*	Kimchi (Korean pickle)	-
#Ec-2	*Enterococcus casseliflavus*	Kimchi (Korean pickle)	-
#Ec-3	*Enterococcus casseliflavus*	Kimchi (Korean pickle)	-
#Efm-1	*Enterococcus faecium*	Soil	-
#Efm-2	*Enterococcus faecium*	Kimchi (Korean pickle)	-
#Eg-2	*Enterococcus gallinarum*	Kidney Bean	-
#Eg-3	*Enterococcus gallinarum*	Kimchi (Korean pickle)	-
#Lcu-1	*Lactobacillus curvatus*	Kimchi (Korean pickle)	-
#Lpb-1	*Lactobacillus parabuchneri*	Kimchi (Korean pickle)	-
#Lp-2	*Lactobacillus paraplantarum*	Kimchi (Korean pickle)	-
#Ls-3	*Lactobacillus sakei*	Kimchi (Korean pickle)	-
#Ls-4	*Lactobacillus sakei*	Kimchi (Korean pickle)	-
#Ls-5	*Lactobacillus sakei*	Kimchi (Korean pickle)	-
#Ls-6	*Lactobacillus sakei*	Kimchi (Korean pickle)	-
#Ll-6	*Lactococcus lactis*	Grape	-
#Ll-7	*Lactococcus lactis*	Bell pepper	+
#Ll-8	*Lactococcus lactis*	Squash	-
#Ll-9	*Lactococcus lactis*	Cherry tomatoes	+
#Lc-2	*Leuconostoc carnosum*	Kimchi (Korean pickle)	-
#Lci-1	*Leuconostoc citreum*	Kimchi (Korean pickle)	-
#Lci-2	*Leuconostoc citreum*	Kimchi (Korean pickle)	-
#Lci-3	*Leuconostoc citreum*	Pickles	-
#Lci-4	*Leuconostoc citreum*	Kimchi (Korean pickle)	-
#Lci-5	*Leuconostoc citreum*	Kimchi (Korean pickle)	-
#Lel-1	*Leuconostoc lactis*	Kimchi (Korean pickle)	-
#Lme-2	*Leuconostoc mesenteroides*	Pear	-
#Lme-3	*Leuconostoc mesenteroides*	Pear	-
#Lme-4	*Leuconostoc mesenteroides*	Pear	-
#Lme-5	*Leuconostoc mesenteroides*	Kimchi (Korean pickle)	-
#Lme-6	*Leuconostoc mesenteroides*	Pickles	-
#Lme-7	*Leuconostoc mesenteroides*	Pickles	-
#Lme-8	*Leuconostoc mesenteroides*	Kimchi (Korean pickle)	-
#Lme-9	*Leuconostoc mesenteroides*	Kimchi (Korean pickle)	-
#Lme-10	*Leuconostoc mesenteroides*	Kimchi (Korean pickle)	-
#Lme-11	*Leuconostoc mesenteroides*	Kimchi (Korean pickle)	-
#Lme-12	*Leuconostoc mesenteroides*	Kimchi (Korean pickle)	-
#Leup-1	*Leuconostoc pseudomesenteroidetes*	Bell pepper	-
#Sw-1	*Staphylococcus warneri*	Kimchi (Korean pickle)	-

#Wc-1	*Weissella cibaria*	Kimchi (Korean pickle)	-
#Wc-2	*Weissella cibaria*	Kimchi (Korean pickle)	-
#Wc-3	*Weissella cibaria*	Kimchi (Korean pickle)	-
#Wc-4	*Weissella cibaria*	Kimchi (Korean pickle)	-
#Wc-5	*Weissella cibaria*	Kimchi (Korean pickle)	-
#Wc-6	*Weissella cibaria*	Kimchi (Korean pickle)	-
#Wco-1	*Weissella confusa*	Kimchi (Korean pickle)	-
#Wh-1	*Weissella hellenica*	Kimchi (Korean pickle)	-
#Wh-2	*Weissella hellenica*	Kimchi (Korean pickle)	-
#Wh-3	*Weissella hellenica*	Kimchi (Korean pickle)	-
#Wk-1	*Weissella koreensis*	Kimchi (Korean pickle)	-
#Wk-2	*Weissella koreensis*	Kimchi (Korean pickle)	-
#Ws-1	*Weissella soli*	Kimchi (Korean pickle)	-

[a]Fifth instar larva were fed a 10% sucrose diet containing various lactic acid bacteria (12.5% in the diet; artificial food of cell wet weight 125 mg and wet weight 875 mg mixed) for 1 h and the hemolymph glucose levels in silkworms were measured. Lactic acid bacteria that significantly decreased the hemolymph glucose level in silkworms (P <0.05) compared with the no-lactic acid bacteria control are indicated by “+” in the far right column.

Supplementary Table 2 | Growth characteristics of the *Enterococcus faecalis* YM0831.

Carbohydrate	YM0831	Carbohydrate	YM0831
Glycerol	±	Salicin	+
Erythritol	−	Cellobiose	+
D-Arabinose	−	Maltose	+
L-Arabinose	−	Lactose	−
Ribose	+	Melibiose	+
D-Xylose	−	Saccharose	+
L-Xylose	−	Trehalose	±
Adonitol	−	Inulin	−
β-Methyl-xyloside	−	D-Melezitose	+
Galactose	+	D-Raffinose	−
D-Glucose	+	Starch	−
D-Fructose	+	Glycogen	−
D-Mannose	+	Xylitol	−
L-Sorbose	−	β-Gentiobiose	+
Rhamnose	−	D-Turanose	±
Dulcitol	−	D-Lyxose	−
Inositol	−	D-Tagatose	+
Mannitol	±	D-Fucose	−
Sorbitol	+	L-Fucose	−
α-Methyl-D-mannoside	−	D-Arabitol	−
α-Methyl-D-glucoside	−	L-Arabitol	−
N-Acetyl glucosamine	+	Gluconate	−
Amygdalin	+	2-Keto-gluconate	−
Arbutin	+	5-Keto-gluconate	−
Esculin	+		

Carbohydrate metabolizing abilities of *Enterococcus faecalis* YM0831 were determined using an API 50 CH kit (Sysmex Corporation, Hyogo, Japan). A colony of *Enterococcus faecalis* YM0831 was suspended in Suspension Medium (Sysmex Corporation) and prepared to McFarland turbidity 2. The bacterial sample (150 μl) was added to an API plate (Sysmex Corporation), and cultured at 30°C for 48 h. The ability to metabolize carbohydrate was determined based on a change in the color of the culture media.

Supplementary Table 3 | Enzyme activities of the *Enterococcus faecalis* YM0831.

Enzyme	YM0831
Alkaline phosphatase	+
Esterase (C4)	+
Esterase Lipase (C8)	+
Lipase (C14)	−
Leucinearylamidase	+
Valinearylamidase	−
Cysteinearylamidase	−
Trypsin	−
α-chimotrypsin	+
Acid phosphatase	+
Naphthol-AS-BI- phosphohydrolase	+
α-galactosidase	−
β-galactosidase	−
β-glucuronidase	−
α-glucosidase	+
β-glucosidase	−
N-acetyl-β-glucosaminidase	−
α-mannosidase	−
α-flucosidase	−

Enzymatic abilities of *Enterococcus faecalis* YM0831 were determined using an API ZYM (Sysmex Corporation, Hyogo, Japan). A colony of *Enterococcus faecalis* YM0831 was suspended in Suspension Medium (Sysmex Corporation) and prepared to McFarland turbidity 5. The bacterial sample (65 μl) was added to an API ZYM plate (Sysmex Corporation), and cultured at 30°C for 4 h. After incubation, ZYM A reagent (1 drop) and ZYM B reagent (1 drop; Sysmex Corporation) were added to the plate. The ability of the enzyme was determined based on a change in the color of the reaction solution.

Supplementary Table 4 | Baseline participant characteristics.

Characteristic	Participants related in Figure 5 (*n* = 14)	Participants related in Supplementary Figure 6 (*n* = 12)	Participants related in Figure 6 (*n* = 10)
Female sex, no. (%)	0 (0)	0 (0)	3 (30)
Age, years	42.6 ± 8.6	44.8 ± 7.5	32.9 ± 6.2
BMI, kg/m^2	22.2 ± 3.1	20.8 ± 3.1	21.6 ± 3.2
AST (GOT), U/L	25.3 ± 10.9	20.3 ± 3.5	19.1 ± 5.3
ALT (GPT), U/L	22.6 ± 12.6	18.4 ± 7.6	15.7 ± 5.5
γGTP, U/L	48.0 ± 50	37.8 ± 21.2	17.6 ± 7.7
Creatinine, mg/dL	0.81 ± 0.13	0.74 ± 0.14	0.68 ± 0.13
Urea nitrogen (BUN), mg/dL	13.6 ± 3.0	13.1 ± 4.1	15.0 ± 2.0
Urea (UA), mg/dL	5.5 ± 1.5	4.7 ± 1.0	5.0 ± 0.8
White blood cell count (WBC), $10^3/\mu L$	6.2 ± 1.6	5.0 ± 1.5	6.1 ± 1.4
Red blood cell count (RBC), $10^4/\mu L$	488 ± 46	486 ± 38	487 ± 30
Hemoglobin (Hb), g/dL	15.2 ± 1.2	15.5 ± 0.9	14.8 ± 0.9
Hematocrit (Ht), %	44.0 ± 2.6	45.3 ± 2.7	44.5 ± 2.3
Platelet count, $10^4/\mu L$	25.7 ± 4.3	19.9 ± 4.5	22.0 ± 3.4

Plus-minus values are means ± SD. The BMI is the weight in kilograms divided by the square of the height in meters.

Supplementary Table 5 | Primers used in this study.

Primer name	Sequences
**RT-PCR**	
manX1-F	CCTGGTGTCAAAGTAAATGTCATCA
manX1-R	CGTCCAAAGGATTAGTGAATAACAAC
manX2-F	CAAAAGCTGCTGCGACAGAA
manX2-R	TTTGCCATCGCCAACAACT
manY-F	ACTTTACAAATGATCGCGTTAGGTT
manY-R	TTGCTGAGGCAACAGATGCT
manZ-F	CCTTGGTGTAACATTAGCTTTGGA
manZ-R	CCTTGAATCGCTACGTCATCAA
manO-F	TCCAAATTCCGTGGGAAGAA
manO-R	TCGCATAACGAGGAATCCATT
EF0025-F	TTCCAAAATCCTGCCGGTAA
EF0025-R	GCATAAGCTTTGTAGCCTAGCAATT
**Plasmid construction**	
man operon-F	ACCCGGGGATCCTCTGCTCTTAATATGGATATTCCAGCAGAAGAA
man operon-R	CTGCAGGTCGACTCTAAAATTTTAGTTCCCGTAGCGATTATCGTG
pND50infusion-F	AGAGTCGACCTGCAGGCATGCAAGCTT
pND50infusion-R	AGAGGATCCCCGGGTACCGAGCTC
