# Supplementary material for: Enterococcus faecalis YM0831 suppresses sucrose-induced hyperglycemia in a silkworm model and in humans
Source: Commun Biol. 2019 May 2;2:157. doi: 10.1038/s42003-019-0407-5 (PMC6497652; doi:10.1038/s42003-019-0407-5)
Supplement: Supplementary file 2 — Description of Additional Supplementary Files [file 42003_2019_407_MOESM2_ESM.docx]

**Description of Additional Supplementary Files**

**File Name**: Supplementary Data 1

**Description**: The file includes dataset of Figures.
