# Supplementary material for: Enterococcus faecalis YM0831 suppresses sucrose-induced hyperglycemia in a silkworm model and in humans
Source: Commun Biol. 2019 May 2;2:157. doi: 10.1038/s42003-019-0407-5 (PMC6497652; doi:10.1038/s42003-019-0407-5)
Supplement: Supplementary file 3 — Reporting Summary [file 42003_2019_407_MOESM3_ESM.pdf]

nature research

Corresponding author(s): Kazuhisa Sekimizu

Reporting Summary

Nature Research wishes to improve the reproducibility of the work that we publish. This form provides structure for consistency and transparency in reporting. For further information on Nature Research policies, see Authors & Referees and the Editorial Policy Checklist.

Statistical parameters

When statistical analyses are reported, confirm that the following items are present in the relevant location (e.g. figure legend, table legend, main text, or Methods section).

n/a	Confirmed	
☐	☒	The exact sample size (*n*) for each experimental group/condition, given as a discrete number and unit of measurement
☐	☒	An indication of whether measurements were taken from distinct samples or whether the same sample was measured repeatedly
☐	☒	The statistical test(s) used AND whether they are one- or two-sided<br>*Only common tests should be described solely by name; describe more complex techniques in the Methods section.*
☐	☒	A description of all covariates tested
☐	☒	A description of any assumptions or corrections, such as tests of normality and adjustment for multiple comparisons
☐	☒	A full description of the statistics including central tendency (e.g. means) or other basic estimates (e.g. regression coefficient) AND variation (e.g. standard deviation) or associated estimates of uncertainty (e.g. confidence intervals)
☒	☐	For null hypothesis testing, the test statistic (e.g. *F*, *t*, *r*) with confidence intervals, effect sizes, degrees of freedom and *P* value noted<br>*Give P values as exact values whenever suitable.*
☒	☐	For Bayesian analysis, information on the choice of priors and Markov chain Monte Carlo settings
☒	☐	For hierarchical and complex designs, identification of the appropriate level for tests and full reporting of outcomes
☒	☐	Estimates of effect sizes (e.g. Cohen's *d*, Pearson's *r*), indicating how they were calculated
☐	☒	Clearly defined error bars<br>*State explicitly what error bars represent (e.g. SD, SE, CI)*

Our web collection on statistics for biologists may be useful.

Software and code

Policy information about availability of computer code

Data collection	eBURST V3 (eburst.mlst.net/V3/mlst_datasets/),
Data analysis	Image J ver. 1.43u (National Institutes of Health, USA), Clustal Omega (www.ebi.ac.uk/Tools/msa/clustalo/), Simple Phylogeny (www.ebi.ac.uk./Tools/services/web_simple_phylogeny/toolform.ebi), and CLC genomic workbench

For manuscripts utilizing custom algorithms or software that are central to the research but not yet described in published literature, software must be made available to editors/reviewers upon request. We strongly encourage code deposition in a community repository (e.g. GitHub). See the Nature Research guidelines for submitting code & software for further information.

Data

Policy information about availability of data

All manuscripts must include a data availability statement. This statement should provide the following information, where applicable:

- Accession codes, unique identifiers, or web links for publicly available datasets
- A list of figures that have associated raw data
- A description of any restrictions on data availability

All data generated or analysed during this study are included in this published article (and its supplementary information files).
Human clinical studies were registered at the Japan UMIN Clinical Trials Registry (UMIN000024338 for Fig. 5 and Supplementary Fig. 4, and UMIN000020807 for

Fig. 6).
DNA sequence of rRNA have been deposited in Genbank (accession number; MK182799).
This Whole Genome Shotgun project has been deposited at DDBJ/ENA/GenBank under the accession SJRZ00000000. The version described in this paper is version DDBJ/ENA/GenBank under the accession SJRZ00000000.
The E. faecalis YM0831 (0831-07) has been deposited in National Institute of Technology and Evaluation (Deposit number: P-02309, NITE, Tokyo, Japan).
Plasmid (pMan operon) have been deposited in Addgene (Deposit number; 76594).

Field-specific reporting

Please select the best fit for your research. If you are not sure, read the appropriate sections before making your selection.

☒ Life sciences ☐ Behavioural & social sciences ☐ Ecological, evolutionary & environmental sciences

For a reference copy of the document with all sections, see nature.com/authors/policies/ReportingSummary-flat.pdf

Life sciences study design

All studies must disclose on these points even when the disclosure is negative.

Sample size	More than three samples. The data are shown as the mean ± SEM. Unless otherwise noted, the significance of differences was calculated using a two-tailed Student's t-test at the significance level P < 0.05.
Data exclusions	No data were excluded from the analyses.
Replication	The reproducibility of the experimental findings were checked. Human clinical studies were not checked the reproducibility.
Randomization	Random
Blinding	The investigator (Dr. Setsuo Hasegawa, MD) and doctors in Osaki Hospital Tokyo Heart Center, Tokyo, Japan was blinded.

Reporting for specific materials, systems and methods

Materials & experimental systems

n/a	Involved in the study	
☒	☐	Unique biological materials
☒	☐	Antibodies
☐	☒	Eukaryotic cell lines
☒	☐	Palaeontology
☐	☒	Animals and other organisms
☐	☒	Human research participants

Methods

n/a	Involved in the study	
☒	☐	ChIP-seq
☒	☐	Flow cytometry
☒	☐	MRI-based neuroimaging

Eukaryotic cell lines

Policy information about cell lines

Cell line source(s)	Caco-2 cells
Authentication	American Type Cell Collection (ATCC, Manassas, VA, USA)
Mycoplasma contamination	Caco-2 cells were tested negative for mycoplasma contamination by the ATCC.
Commonly misidentified lines (See ICLAC register)	*Name any commonly misidentified cell lines used in the study and provide a rationale for their use.*

Animals and other organisms

Policy information about studies involving animals; ARRIVE guidelines recommended for reporting animal research

Laboratory animals	Silkworms. Fertilized silkworm eggs (Bombyx mori, Hu · Yo x Tukuba · Ne) were purchased from Ehime sericulture incorporated company (Ehime, Japan).

Wild animals	*Provide details on animals observed in or captured in the field; report species, sex and age where possible. Describe how animals were caught and transported and what happened to captive animals after the study (if killed, explain why and describe method; if released, say where and when) OR state that the study did not involve wild animals.*
Field-collected samples	*For laboratory work with field-collected samples, describe all relevant parameters such as housing, maintenance, temperature, photoperiod and end-of-experiment protocol OR state that the study did not involve samples collected from the field.*

Human research participants

Policy information about studies involving human research participants

Population characteristics	Population characteristics were described in supplementary Table 4.
Recruitment	Participants were randomly recruited at Osaki Hospital Tokyo Heart Center, Tokyo, Japan, and all subjects provided written informed consent before initiation of any study procedures.
